# The effect of giving influenza vaccination to general practitioners: a controlled trial [NCT00221676]

**DOI:** 10.1186/1741-7015-4-17

**Published:** 2006-07-10

**Authors:** Michiels Barbara, Philips Hilde, Coenen Samuel, Yane Fernande, Steinhauser Toon, Stuyck Sofie, Denekens Joke, Van Royen Paul

**Affiliations:** 1 Department of Family Medicine, Centre for General Practice, University of Antwerp, University of Antwerp – Campus Drie Eiken, Universiteitsplein 1, 2610 Antwerp, Belgium; 2 Fund for Scientific Research – Flanders, Brussels, Belgium; 3 National Influenza Centre, IPH – Scientific Institute of Public Health, 14 rueJuliette Wytsman, B-1050 Brussels, Belgium

## Abstract

**Background:**

No efficacy studies of influenza vaccination given to GPs have yet been published. Therefore, our purpose was to assess the effect of an inactivated influenza vaccine given to GPs on the rate of clinical respiratory tract infections (RTIs) and proven influenza cases (influenza positive nose and throat swabs and a 4-fold titre rise), while adjusting for important covariates.

**Methods:**

In a controlled trial during two consecutive winter periods (2002–2003 and 2003–2004) we compared (77 and 100) vaccinated with (45 and 40) unvaccinated GPs working in Flanders, Belgium. Influenza antibodies were measured immediately prior to and 3–5 weeks after vaccination, as well as after the influenza epidemic. During the influenza epidemic, GPs had to record their contact with influenza cases and their own RTI symptoms every day. If they became ill, the GPs had to take nose and throat swabs during the first 4 days. We performed a multivariate regression analysis for covariates using Generalized Estimating Equations.

**Results:**

One half of the GPs (vaccinated or not) developed an RTI during the 2 influenza epidemics. During the two influenza periods, 8.6% of the vaccinated and 14.7% of the unvaccinated GPs had positive swabs for influenza (RR: 0.59; 95%CI: 0.28 – 1.24). Multivariate analysis revealed that influenza vaccination prevented RTIs and swab-positive influenza only among young GPs (ORadj: 0.35; 95%CI: 0.13 – 0.96 and 0.1; 0.01 – 0.75 respectively for 30-year-old GPs). Independent of vaccination, a low basic antibody titre against influenza (ORadj 0.57; 95%CI: 0.37 – 0.89) and the presence of influenza cases in the family (ORadj 9.24; 95%CI: 2.91 – 29) were highly predictive of an episode of swab-positive influenza.

**Conclusion:**

Influenza vaccination was shown to protect against proven influenza among young GPs. GPs, vaccinated or not, who are very vulnerable to influenza are those who have a low basic immunity against influenza and, in particular, those who have family members who develop influenza.

## Background

There are two important issues when considering influenza vaccination of general practitioners (GPs) as advocated by many guidelines. [1,2] Firstly, an influenza vaccine must give personal protection to the GP. To a certain extent, this issue has been addressed by efficacy studies among healthy adults. [3] Secondly, vaccination might be useful for preventing transmission of influenza between GPs and their patients. For example, in long-term care hospitals, influenza vaccination of healthcare workers reduced mortality among the elderly. [4,5]However, owing to the low basic immunity against influenza among healthy adults and healthcare workers working in long-term care facilities, the results of these studies are not fully applicable to general practice.

Since GPs have frequent close contact with many influenza cases, they build up a high basic immunity and probably only suffer from minor symptoms. [6,7]Whether the vaccine adds substantial benefit to this naturally acquired immunity is unknown. Inactivated vaccines are not very useful in preventing cross-infection and the shedding of viruses from the nose and throat; [8,9]they are only known to diminish the severity of the influenza symptoms and to prevent complications, especially when compared to intra-nasally administered influenza vaccines (inactivated whole virus, [10]with adjuvants, [11]or live cold-adapted) [9]that elicit a better local immune response (mucosal IgA) in the nose, throat and airways. Unfortunately, these new vaccines are not yet commercially available in Europe.

Until now, no efficacy studies of influenza vaccination among GPs have been published. Therefore, our purpose was to assess the effect of an inactivated influenza vaccine given to GPs on clinical respiratory tract infections (RTIs) and, more particularly, against influenza cases with influenza-positive nose and throat swabs (diagnosed by reverse transcriptase polymerase chain reaction RT-PCR), in addition to serologically-defined influenza cases. We also adjusted for relevant covariates.

## Methods

### 1. Design of the study

A controlled trial during two consecutive winter periods (2002–2003 and 2003–2004) was performed, comparing vaccinated and unvaccinated GPs working in Flanders recruited on a voluntary basis in July and August 2002 and 2003. First-year participants were asked to re-enter the study during the second winter period. Subjects were enrolled after giving their written informed consent. The study was approved by the Medical Ethics Committee of the University Clinic of Antwerp. Participating GPs had to fill in a questionnaire relating to their general characteristics and previous influenza vaccinations. Owing to ethical considerations, the GPs were free to choose whether or not to receive an influenza vaccination during the study period. Those who wanted to be vaccinated were instructed to have the 0.5-ml vaccine administered into the deltoid muscle, at the end of October of each study year. GlaxoSmithKline n.v. provided Alfarix^®^, a commercially available non-adjuvant trivalent inactivated split-influenza vaccine, to each participating GP personally for this study. In 2002 – 2003 and 2003 – 2004 the vaccine contained the same strains: 15 μg hemagglutinin from A/New Caledonia/20/99 (H1N1), A/Moscow/10/99 (= A/Panama/2007/99) (H3N2) and B/Hong Kong/330/2001.

### 2. Blood collection and serology

Blood specimens for the antibody studies were taken immediately prior to and 3–5 weeks after vaccination. Unvaccinated GPs only gave 1 blood specimen in November before the influenza epidemic, assuming this would give the same antibody titres as blood samples taken one month earlier (= pre-epidemic).

Three weeks after the influenza epidemic, both groups gave another blood specimen (= post-epidemic). The blood samples were collected by local medical laboratories for serum extraction and preservation (-20°C). After the last blood sample was taken, the serum tubes were transported to Viroclinics in Rotterdam.

Viroclinics did a hemagglutination-inhibition (HAI) test using standard methods to measure the influenza antibody titre against the circulating influenza strains. In 2002–2003, antibody titres were determined against A/H1N1 (IVR-116), A/H3N2 (ResVir-17) and B/Shangdong/7/'97. In 2003–2004, only one strain was circulating and therefore only the titre against A/H3N2 (Fujian-like) was measured. Each test was performed twice and the average titre was taken as the outcome. Laboratory personnel were blinded to the identity and study group of the serum samples. We considered a 4-fold or greater titre rise detected in the paired samples collected at any time between pre- and post-epidemic periods as indicative of an immune reaction following an influenza virus infection.

### 3. Daily diary recording

For 60 consecutive days (weekends and holidays included), the GPs were asked to complete diaries.

**Figure 1:**
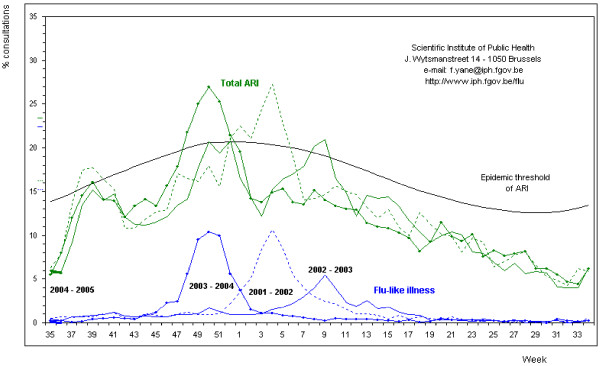
The incidence of influenza-like illnesses (ILI) and acute respiratory infections (ARI) recorded by sentinel physicians in Belgium.

Instruction to start daily diary entries was given as soon as the Scientific Institute of Public Health (IPH – ) in Brussels detected the first influenza viruses in nose and throat swabs collected by sentinel physicians in Belgium during two successive weeks (Figure 1). In the 2002–2003 winter period, diary recording started on February 13, 2003 (7^th ^week); in the 2003–2004 winter period, diary recording was started much earlier, on November 22, 2003 (47^th ^week).

The GPs were asked to record the number of contacts with patients in general and with those diagnosed as influenza cases (patients, family and other contacts) in particular. The GPsalso had to report the number of influenza patients whomthey hadseenfor another reason within a week prior to having diagnosedthe patients with influenza. An influenza case was defined as any person for whom the GP established a clinical diagnosis of influenza on the basis of the following symptoms and signs: sudden onset, high fever (above 38°C), cough and myalgia.

GPs suffering from the slightest symptom of an RTI had to mention this in their diaries and record their oral body temperature, and also to record the severity of their own signs and symptoms. Furthermore, affected GPs had to indicate their diagnosis, their medication, and any sick leave taken.

After the recording periods ended, the diaries were sent by post (2002–2003) or collected personally (2003–2004).

### 4. Influenza virus detection

GPs were asked to brush their nose with two cotton swabs and their throat with 1 cotton swab from the first to the fourth day during each RTI that they developed during the recording period. The swabs were immersed in a transport medium (Earle's minimal essential medium (EMEM) with addition of antibiotics and antimycotic products) and were stored by the local medical laboratory where the samples were collected by a courier of the Scientific Institute of Public Health (IPH). After the influenza epidemic, the transport media were analysed with a nested RT-PCR test first to distinguish between A and B strains, and then to subtype the A strains as H3 or H1. Uncertain results were retested. Laboratory personnel were blinded to the identity and study group of the swabs.

### 5. Statistics

A preliminary sample size estimation was performed using Epi-info. If we assumed a clinically significant vaccine efficacy of 70% in preventing influenza and an incidence of serologically-defined influenza in the unvaccinated group of 23%, [7]or an incidence as defined by virus detection (RT-PCR) of 8.5% (result of a pilot study), then we needed 124 and 368 vaccinated GPs and 62 and 184 unvaccinated GPs respectively (a 2:1 ratio) for a power of 80% and a two-sided significance level of 0.05.

Differences in proportions were assessed by Pearson χ^2 ^tests and odds ratios (95%CI); continuous variables were analysed by Student's *t*-tests after converting serum titres to the natural logarithm using SPSS version 12.0.

We performed a multivariate regression analysis for relevant covariates using Generalized Estimating Equations (GEE) (GENMOD in SAS statistics version 8.02) following the Hierarchical Backward Elimination Approach of Kleinbaum [12]to determine which factors other than vaccination influence the three dichotomous outcomes defined as RTIs in general, RTIs with influenza-positive nose and throat swabs, and RTIs with influenza-positive nose and throat swabs and/or a 4-fold titre rise. Confounding variables of interest included: study year; age in years; basic immunity (lnT1); mean number of patient contacts/day; mean number of influenza patients/day; family members with influenza; colleagues with influenza; having young children (<7 years); and working in a Child and Family preventive medicine facility (Table 3). In addition, significant interaction terms between vaccination and these confounding variables were introduced in the model.

## Results

### 1. General characteristics

**Figure 2:**
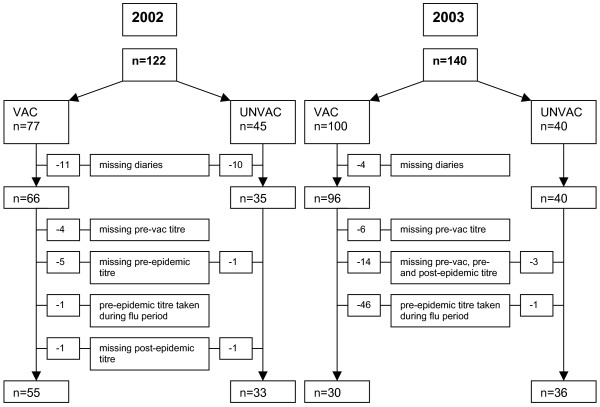
Flowchart of GPs' missing diaries and serology data.

A total of 122 GPs, of whom 77 were vaccinated against influenza in October 2002, were included in the first part of the study, while 140 GPs, of whom 100 were vaccinated in October 2003, participated in the second part. Overall, 72 GPs participated in both parts. Missing and eliminated data (diaries and serology) are shown in Figure 2.

In 2003, we had to consider 47 (46 vaccinated) second serum samples to be missing because these were taken during the influenza epidemic that started as early as November 22, 2003. Thirteen sick GPs in 2002 and 11 sick GPs in 2003 did not take nose and throat swabs as requested.

Table 1 shows that most characteristics did not differ between the vaccinated and unvaccinated groups. There were fewer patient contacts in the unvaccinated group: in 2002 and 2003, 21 and 20 patients/day in the vaccinated group versus 18 and 17 patients/day in the unvaccinated group, respectively (p < 0.05). As expected, the vaccinated group was also highly vaccinated against influenza in previous years: more than 80% versus less than 20% in the unvaccinated group (p < 0.0001). Consequently, in the first serum sample, higher geometric mean serum antibody titres (GMT) were measured in the vaccinated group: in 2002, 125 versus 77 (p < 0.05); in 2003, 54 versus 22 (p < 0.0001). The vaccinated GPs also had a higher seroprotection level (number of GPs with a baseline titre > 40): in 2002, 91% versus 80% (NS); in 2003, 70% versus 42% (p < 0.05).

### 2. Efficacy and effectiveness of influenza vaccination

In 2002 and 2003, more than half the GPs suffered at least once from an RTI (mild infections included). Overall, influenza vaccination of GPs had no efficacy in preventing RTIs in general in 2002 or 2003 (Table 2). In 2002, only about 5% of vaccinated GPs and 6% of unvaccinated GPs had positive swabs. However, in 2003, 12% of vaccinated GPs had positive swabs compared with 23% of unvaccinated GPs. The efficacy (= 100(1 – RR)) of vaccination in preventing positive nose and throat swabs showed an increasing trend from 22% in 2002 to 49% in 2003, but this trend was not statistically significant, even when both years (41%) were combined. When the results of both years were combined, vaccination had a significant effect on the 4-fold titre rise alone (72%; 95%CI: 25–90%) and in combination with positive swabs (56%; 95%CI: 12–78%). For these comparisons, possible confounders were not taken into account.

The mean of the highest body temperature of every RTI episode was 37.4°C in the influenza infections (positive swabs); this was statistically significantly higher than during the other RTIs (p = 0.005). In general, the influenza infections lasted 2 days longer (p = 0.03). Of the GPs with positive swabs, 20% took sick leave, compared to 12% of GPs suffering from other RTIs (p = 0.3).

### 3. Multivariate analysis

**Figure 3:**
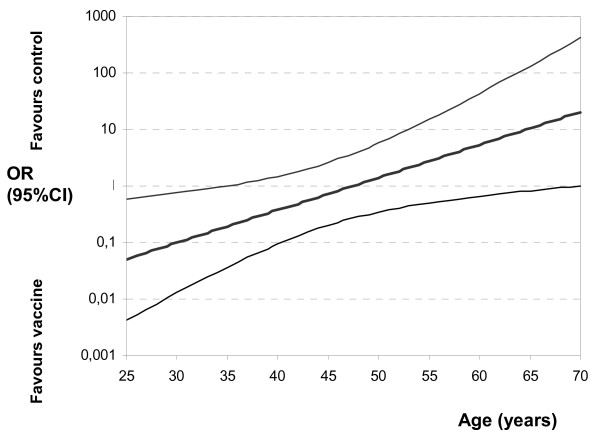
The effect of influenza vaccination (OR) on respiratory tract infections with influenza-positive nose and throat swabs for different ages: multivariate regression analysis.

Table 3 shows the results of the multivariate regression analysis for correlated data for the 3 dichotomous outcomes defined as RTIs in general, RTIs with influenza-positive nose and throat swabs, and RTIs with influenza-positive nose and throat swabs and/or a 4-fold titre rise. For the first two outcomes, vaccine*age was the only significant interaction term (Wald p < 0.02). Therefore, this interaction term yielded different effect sizes of influenza vaccination for different age groups. For example, influenza vaccination of 30-year-old GPs was effective, independent of the definition of the outcome. At this age, the efficacy of the vaccine in preventing positive nose and throat swabs was as high as 90%. However, at 50 years of age, influenza vaccination had no effect (OR>1), except in preventing influenza cases defined as positive swabs and/or a 4-fold titre rise. For preventing proven swab-positive influenza, the OR was statistically significant under 36 years of age (OR 0.22; 95%CI: 0.04 – 1.06) and became 1 at 48 years of age (Figure 3). Other than vaccination, no other variable had an important effect on RTIs in general. More specifically, the prevention of positive swabs and/or a 4-fold titre rise depended on the basic immunity against influenza reflected in this model by the natural logarithm of the A/H3N2 influenza antibody titre of the first serum sample (OR for a difference of 1 in lnT1 = 0.57; 95%CI: 0.38 – 0.86). In addition, the presence of family members with influenza was highly predictive for retrieving positive nose and throat swabs and/or a 4-fold titre rise (OR = 3.62; 95%CI: 1.20 – 10.89). Careful review of the diary data revealed that 20 of 25 PCR-proven influenza cases had a family member who was also affected. In 13 cases, the family member was ill prior to the GP, and in 3 cases concurrently with the GP.

## Discussion

Multivariate analysis revealed that influenza vaccination prevented both respiratory tract infections in general and proven influenza during an influenza epidemic, especially among young general practitioners. Independently of vaccination, a low basic antibody titre against influenza and the presence of influenza cases in the family were highly predictive of an episode of influenza with positive swabs and/or a 4-fold titre rise.

During the recording periods in both years, half the GPs had some form of RTI. That 50% of GPs were affected by RTIs seems rather high, but could be explained by the fact that even mild symptoms were recorded and reflects the occurrence of mild infections through which natural immunity is constantly updated. The general RTI definition used in this study was too imprecise to allow a significant vaccination effect to be shown.

Our study was slightly underpowered for demonstrating statistically significant efficacy of an influenza vaccination in preventing RTIs with RT-PCR-positive nose and throat swabs. There was a large difference in efficacy between the two years: 22% in 2002 and 49% in 2003. Furthermore, in the unvaccinated group, the incidence of positive influenza cases rose from 5% in 2002 to 23% in 2003. During the first study period (2002), there was a high basic immunity against influenza A as the cumulative result of the previous 2 years in which the circulating influenza virus A strains did not change much. [13]According to the IPH, the peak incidence of influenza-like illnesses in the community was 5% in week 9 in the first study year and 10% in week 50 of the following influenza season (2003–2004) [14](Figure 1). During 2003/2004, a slightly different influenza A virus was circulating. Although this new A/Fujian-like A/H3N2 virus was different from the A strains contained in the vaccine, the vaccine elicited a good immune response. [15,16]An epidemiological study performed among GPs during the Asian influenza epidemic of 1957 concluded that, in many parts of Britain, doctors were exposed to a risk of influenza at least double that of the general population in their practices. [17]

The RT-PCR test is more sensitive than a virus culture, [4,18]since it also detects dead virus particles and is not quantitative. Therefore, no conclusions can be drawn about the degree of infectivity of the GPs with positive nose and throat swabs. No significant difference was seen between the GPs with infectious influenza and the other GPs with RTIs with respect to comparing the number of patients diagnosed with influenza and seen by the GP 1 week before for another reason, and 14 days before and after the GP became ill.

This study confirmed the efficacy of influenza vaccination on serologically-defined influenza; the efficacy of influenza vaccination was 72% for the 2 years combined, which is comparable with an efficacy of 88% (95%CI: 47–97%) in preventing a 4-fold titre rise against A/H3N2 reported by Wilde et al. [19]Since no studies of the efficacy of influenza vaccination among GPs have previously been published, we can only compare our results with trials performed among other healthcare professionals working in hospitals. A Cochrane review dealing with vaccines given to prevent influenza in healthy adults included the results of trials involving healthcare workers. [3]None of these studies demonstrated significant efficacy of influenza vaccination on influenza cases (clinically defined and sometimes serologically confirmed), and none of the studies presented results that evaluated the ability of vaccination to interrupt the spread of the disease (see [additional file 1]: existing literature concerning our study results.)

Multivariate analysis revealed that the effect of influenza vaccine decreased with age. Young GPs have a higher incidence of influenza, and they have families with young children. [20]Although these findings need to be confirmed by larger studies, they suggest that young GPs should be the first to be vaccinated against influenza. Medical students and postgraduate trainees would also benefit from being among the first to be vaccinated.

However, the question arises as to whether influenza vaccination can be stopped at a certain age. It is likely that GPs who have been working for more than 20 years in full-time practice and who have enough yearly contact with influenza patients do not need to be vaccinated. Healthcare workers with fewer patient contacts need influenza vaccination throughout their entire career. [21]These considerations are only applicable in the inter-pandemic period. When a new virus emerges, such as an adapted avian influenza virus, and then when an appropriate vaccine is available, all healthcare workers, without exception, would be a priority group for vaccination.

The level of anti-hemagglutinin antibody titres before vaccination is another independent protective variable that has been known for quite some time. [22]It was relatively high in our GP population and reflects the cumulative effect of previous vaccinations and natural infections. [16]In our study, age was not related to the antibody titre levels before the influenza epidemic. In addition to serological immunity, other immune factors, such as cellular [23]and local mucosal immunity, which were not measured in this study, may play an important role. [10]

Another underestimated factor is the infectiousness of family members with influenza. Independently of age or vaccination status, sick family members, especially children, were found to be very contagious. This result is not new, [24,25]but the consequences have been minimized. Among GPs during the 1957 Asian influenza epidemic, it was noted that 9 of 19 GPs with a typical influenza attack had a family member who had influenza. The authors concluded that GPs were liable to be infected with influenza at home, even after avoiding infection for several weeks during repeated daily contact with patients who have influenza. [17]Neuraminidase-inhibitors could be taken preventively by healthcare workers who have a family member who most probably has influenza during an influenza epidemic. [26]Treating GPs only when they already have influenza is not useful, as the signs and symptoms will most likely be mild and non-specific, making the diagnosis of influenza difficult. However, the use of neuraminidase-inhibitors has already resulted in resistant virus strains; [27]thus, prevention using a better-designed vaccine is preferable.

Further research is needed, focusing particularly on assessing the efficacy of new vaccines for use among healthcare workers in preveningt influenza virus growth in the nose and throat.

Some limitations of this study need to be considered. It was underpowered for detecting a significant effect of influenza vaccination on influenza cases with positive nose and throat swabs; to detect an efficacy of 70% with a measured incidence of proven influenza of 14.7% in the unvaccinated group, at least 208 vaccinated and 104 unvaccinated GPs would have been needed. However, it remains to be determined whether the efficacy in healthcare workers need not be higher to prevent possible infectious cases of influenza and protect against transmission to patients. If we assume an efficacy of 90% as clinically relevant, we only needed 108 vaccinated and 54 unvaccinated GPs to participate in our study.

Unfortunately, the influenza epidemic started very early in the second study year (November 22, 2003). Therefore, 47 serum samples taken after vaccination (T2) during this epidemic were classified as missing. Titres measured in these samples were significantly higher than those taken before November 22, 2003. There were no differences between the GPs with missing serum samples and those used in the analysis, except that fewer GPs with missing serology had allergies; however, since allergy was not related to the incidence of RTIs or influenza, this did not affect the results. The only problem that remains is an underestimation of influenza cases that were defined as a 4-fold titre rise after the epidemic. However, this definition of an influenza case remains questionable, especially in a study group with high antibody titres before the influenza epidemic. Since the unvaccinated group started with a lower antibody titre, a greater number of 4-fold titre rise cases could be expected in this group. Other authors [7,19,28,29]have already mentioned this disproportion and have noted that it is of no clinical importance.

In this article we did not focus on the immunogenetic properties of the vaccine, [16]because knowing the efficacy of the vaccine on clinical outcomes after an epidemic challenge is far more appropriate for policy development. Although the GPs in our study were not chosen at random, we believe the results are representative. In fact, the average age of the participants did not differ from national averages. In 2003, slightly more women than average participated in our study, but this was taken into account in the multivariate analysis.

It is possible that a selection bias was introduced by having the GPs themselves choose whether to be vaccinated. Indeed, the most important difference between vaccinated and unvaccinated GPs was the number of influenza vaccinations in previous years and their effect on basic immunity. However, this too was taken into account in the multivariate analysis.

## Conclusion

Among general practitioners, the efficacy of an influenza vaccination against proven influenza (virological and/or serological defined) was rather moderate in this clinical trial performed during two consecutive winter periods (2002–2003); however, the efficacy was substantially higher among younger GPs. Thus, younger GPs should be the first to be vaccinated. Family members with influenza, rather than the daily burden of influenza patients, make every GP, vaccinated or not, very vulnerable to infectious influenza. Every GP should be aware of this route of infection and should take adequate precautions.

## Competing interests

The author(s) declare that they have no competing interests.

## Authors' contributions

BM participated in the coordination and the design of the study, in the data collection and management, in the statistical analysis, and in the writing and editing of the manuscript.

HP participated in the coordination and the design of the study, in the data collection and management, and in the editing of the manuscript.

SC participated in the design of the study, in the statistical analysis, and in the writing and editing of the manuscript.

FY participated in the design of the study, in the data collection, in the laboratory analysis, and in the editing of the manuscript.

TS and SS participated in the data collection and management and in the editing of the manuscript.

JD participated in the design of the study and in the supervision and editing of the manuscript.

PVR participated in the coordination and the design of the study, in obtaining funding, in the data collection, in the statistical analysis, and in the supervision and editing of the manuscript.

All authors read and approved the final manuscript.

## Pre-publication history

The pre-publication history for this paper can be accessed here:



## Supplementary Material

Additional File 1 Existing literature concerning our study results. To situate our study results within the existing literature Click here for file

## Figures and Tables

**Table 1:** General characteristics of participating GPs: figures are numbers (percentage) unless stated otherwise.

	**2002**		**2003**	
	
	VAC	UNVAC	VAC	UNVAC
**General**				

Men	50/77 (64.9)	25/45 (55.6)	61/100 (61.0)	18/40 (45.0)
Mean (SE) of age	42.4 (1.1)	40.0 (1.5)	43.1 (0.9)	41.3 (10.8)
Influenza vaccine 1 and 2 years before	60/77 (77.9)	3/43 (7.0) ‡	67/94 (71.3)	3/38 (7.9) ‡
Influenza vaccine 1 year before	66/77 (85.7)	3/44 (6.8) ‡	78/95 (82.1)	3/39 (7.7) ‡
Influenza vaccine 1, 2, and 3 years before	54/71 (76.1)	1/42 (2.4)‡	62/91 (68.1)	3/38 (7.9) ‡
Influenza vaccine 2 years before	64/77 (83.1)	8/43 (18.6) ‡	73/94 (77.7)	3/38 (7.9) ‡
Influenza vaccine 3 years before	59/71 (83.1)	6/42 (14.3) ‡	73/91 (80.2)	4/38 (10.5) ‡
Influenza disease 1 year before	9/75 (12.0)	7/42 (16.7)	11/93 (11.8)	4/38 (10.5)
Working in Child and Family preventive medicine facility	11/75 (14.7)	4/45 (8.9)	13/100 (13.0)	4/40 (10.0)
Having young children < 7 years of age	20/70 (28.6)	14/44 (31.8)	21/98 (21.4)	10/40 (25.0)
Smoking	7/72 (9.7)	3/42 (7.1)	9/96 (9.4)	1/39 (2.6)
Allergy	21/72 (29.2)	9/42 (21.4)	23/94 (24.5)	6/38 (15.8)
				
Seroprotection at baseline^1 ^against A:/H3N2	61/67 (91.0)	31/39 (79.5)	56/80 (70.0)	15/36 (41.7) †
GMT1^2 ^A:/H3N2 (SE)	125.39(1.12)	77.26 (1.19) †	54.25 (1.14)	22.34 (1.23) ‡
				

**Information from the diaries**				

GPs with family members with influenza	34/66 (51.5)	19/35 (54.3)	54/96 (56.3)	20/40 (50.0)
GPs with colleagues with influenza	23/66 (34.8)	12/35 (34.3)	35/96 (36.5)	15/40 (37.5)
Mean (SE) number of patient contacts/day	21.3 (1.0)	17.9 (1.3) †	20.4 (0.8)	16.6 (1.2) †
Mean (SE) number of patients with influenza/day	1.6 (0.2)	1.7 (0.2)	2.0 (0.2)	2.1 (0.3)

^1^number of GPs with a baseline titre (T1) > 40

^2 ^geometric mean baseline titre (T1)

† p-value < 0.05

‡ p-value < 0.0001

**Table 2:** Efficacy and effectiveness of influenza vaccination according to different outcomes: crude results

		VAC	UNVAC	RR	95% Cl
Number of GPs with a respiratory tract infection	2002	37/66 (56.1%)	21/35 (60%)	0.93	0.66 – 1.32
	2003	48/96 (50%)	19/40 (47.5%)	1.05	0.72 – 1.54
	2002–2003	85/162 (52.5%)	40/75 (53.3%)	0.98	0.76 – 1.27
					
Number of GPs with a respiratory tract infection and positive nose and throat swabs	2002	3/66 (4.5%)	2/35 (5.7%)	0.78	0.13 – 4.94
	2003	11/96 (11.5%)	9/40 (22.5%)	0.51	0.23 – 1.13
	2002–2003	14/162 (8.6%)	11/75 (14.7%)	0.59	0.28 – 1.24
					
Number of GPs with a respiratory tract infection and a 4-fold hemagglutination-inhibition antibody titre rise	2002	2/59 (3.4%)	4/33 (12.1%)	0.28	0.05 – 1.45
	2003	3/36 (8.3%)	9/36 (25%)	0.33	0.10 – 1.13
	**2002–2003**	**5/95 (5.3%)**	**13/69 (18.8%)**	**0.28**	**0.10 – 0.75**
					
Number of GPs with a respiratory tract infection and positive swab or 4-fold hemagglutination-inhibition antibody titre rise	2002	4/59 (6.8%)	6/33 (15.2%)	0.45	0.13 – 1.55
	2003	7/36 (19.4%)	13/36 (36.1%)	0.54	0.24 – 1.19
	**2002–2003**	**11/95 (11.6%)**	**18/69 (26.1%)**	**0.44**	**0.22 – 0.88**

**Table 3:** Effect of influenza vaccination on respiratory tract infections (RTIs) in general, with influenza positive swabs and/or 4-fold titre rise: multivariate regression analysis for correlated data (GEE)

**Outcome**	**RTI in general (n = 202)**			**RTI with positive swabs for influenza (n = 202)**			**RTI with pos swabs and/or 4-fold titre rise for influenza (n = 150)**		
Parameter^1^	OR estimate	95%CI		OR estimate	95%CI		OR estimate	95%CI	

		Lower limit	Upper limit		Lower limit	Upper limit		Lower limit	Upper limit

Vaccine^†2 ^when GP is 30 years old	**0.35**	**0.13**	**0.96**	**0.1**	**0.01**	**0.75**	**0.13**	**0.02**	**0.98**
Vaccine† when GP is 50 years old	1.79	0.80	4.00	1.41	0.35	5.76	0.82	0.21	3.25
**Co-variates**									
Year 2002/2003	0.98	0.49	1.96	0.42	0.13	1.42	0.52	0.18	1.53
Age‡^3^	0.49	0.17	1.43	0.58	0.17	1.96	0.37	0.09	1.53
Gender†	0.60	0.33	1.10	0.96	0.29	3.23	0.99	0.33	2.94
Basic immunity (natural logarithm of titre1)‡^4^	1.18	0.91	1.55	**0.57**	**0.37**	**0.89**	**0.57**	**0.38**	**0.86**
Mean number of patient contacts/day‡^5^	1.17	0.50	2.76	1.77	0.47	6.69	3.13	0.69	14.11
Mean number of contacts with influenza patients/day‡^6^	0.67	0.44	1.00	0.33	0.10	1.12	0.39	0.13	1.16
Family members with influenza†	1.88	0.97	3.64	**9.24**	**2.91**	**29.40**	**3.62**	**1.20**	**10.89**
Colleagues with influenza†	1.10	0.60	2.01	0.60	0.19	1.84	0.71	0.25	2.04
Having young children < 7 years of age†	2.00	0.95	4.24	1.37	0.42	4.52	1.04	0.30	3.67
Working in Child and family preventive medicine facility†	0.76	0.31	1.87	1.76	0.52	5.92	1.15	0.28	4.66
									
† dichotomous yes(1), no(0)									
‡ continuous									
^1^Intercept =	0.96	-1.63	3.54	0.68	-2.24	3.61	2.44	-1.41	6.28
^2 ^Crude OR vaccine =	0.97	0.56	1.67	0.55	0.24	1.28	**0.35**	**0.15**	**0.79**
^3 ^OR for difference of 20 years									
^4 ^OR for a difference of 1 in natural logarithm of titre1									
^5 ^OR for a difference of 20 patients									
^6 ^OR for a difference of 2 influenza patients									
