# Supplementary material for: The effect of giving influenza vaccination to general practitioners: a controlled trial [NCT00221676]
Source: BMC Med. 2006 Jul 10;4:17. doi: 10.1186/1741-7015-4-17 (PMC1538610; doi:10.1186/1741-7015-4-17)
Supplement: Additional File 1 — Existing literature concerning our study results. To situate our study results within the existing literature [file 1741-7015-4-17-S1.doc]

**Existing literature concerning our study results**

The Cochrane Database of Systematic Reviews 2005;(3) mentions the protocol of a systematic review about influenza vaccination for healthcare workers who work with the elderly [1]. Results are not yet published.

However, another review with the title “Vaccines for preventing influenza in healthy adults” by Demicheli et al. is perhaps more suitable [2]. Their conclusions are as follows: “Influenza vaccines are effective in reducing serologically confirmed cases of influenza. However, they are not as effective in reducing cases of clinical influenza and number of working days lost. Universal immunisation of healthy adults is not supported by the results of this review” and “None of the studies included in the review presented results evaluating the ability of this vaccination to interrupt the spread of the disease.”

When we look in more detail we notice that a few studies were included with healthcare workers or medical students as participants. Their specifications and results (average treatment effects estimated from random effects models) were presented in the review as follows:

1. Keitel 1988 [3]: double-blind RCT including healthy volunteers, 30 to 60 years old, working in the Texas Medical Center in Houston, Texas, USA or in surrounding industrial companies; compares a trivalent, killed-whole, intramuscularly administered vaccine against placebo; results: no significant result on clinical cases of influenza (non specific definition) (RR 0.90 (0.68 – 1.18)) and (RR 1.06 (0.80 – 1.40))
2. Hammond 1978 [4]: CCT including medical students or staff members at Monash University, Australia; compares trivalent, subunit vaccine with a diphtheria-tetanus vaccine; results: no significant result on clinical cases of influenza (non-specific definition) (RR 1.04 (0.85 – 1.26))
3. Weingarten 1985 [5]: RCT: including healthy volunteer hospital employees, USA, 21 to 65 year olds, compares a split trivalent influenza vaccine with placebo; results: no significant result on clinical cases of influenza (specific definition) (RR 1.07 (0.62 – 1.85))
4. Tannock 1984 [6]: a double-blind, placebo-controlled trial, aged 16 to 64, from Newcastle Hospital and the Commonwealth Steel Corporation, Australia, compares a trivalent subunit vaccine with placebo; results: no significant result on clinical cases of influenza (serologically confirmed) (RR 0.54 (0.04 – 8.19))
5. Wilde 1999 [7]: RCT: health care professionals without chronic medical problems and younger than 50 years, USA, compares trivalent, inactivated influenza vaccine with another vaccine; results: no significant result on clinical cases of influenza (serologically confirmed) (RR 0.14 (0.02 -1.13))

In addition we performed our own search in Medline and the Cochrane Central Register of Controlled Trials by using keywords focusing more on healthcare workers[[1]](#footnote-2)

and the following trials were selected:

1. Saxen H 1999 [8]: placebo-controlled double blind RCT on the efficacy of influenza immunization on absenteeism of health care workers in two pediatric hospitals during the winter season 1996 to 1997; results: immunization failed to reduce episodes of respiratory infections (1.8 episodes/study period among vaccinees vs. 2.0 among controls). Similarly the vaccine failed to affect the total number of days the vaccinees suffered from respiratory infections (13.5 days vs. 14.6 days, respectively). However, days of work lost because of respiratory infections (1.0 days vs. 1.4 days, respectively, P = 0.02) and especially total numbers of days the study persons felt themselves unable to work when either on or off duty (2.5 days vs. 3.5 days, P = 0.02), were significantly decreased.

2. Carman WF 2000 [9]: RCT: assessing effects of influenza vaccination of health-care workers on mortality of elderly people in long-term care (UK); results: vaccination of health-care workers was associated with a substantial decrease in mortality among patients, but became statistically insignificant when adjusted for Barthel score, age, sex and vaccination of patients (OR 0.61 (0.36 – 1.04)). However, virological surveillance showed no associated decrease in non-fatal influenza infection in patients.

3. Potter J 1997 [10]: cluster randomized trial among healthcare workers of geriatric long term care hospitals in Glasgow (UK); results: vaccination of HCWs was associated with reductions in total patient mortality from 17% to 10% (OR 0.56 (0.40-0.80)) and in influenza-like illness (OR 0.57 (0.34-0.94)), an association with virologically proven influenza among patients could not be shown. Vaccination of patients was not associated with significant effects on mortality (OR 1.15(0.81-1.64)).

1. Search keywords used in different combinations

   (healthcare worker.mp. [mp=ti, ot, ab, nm, hw] OR health care worker.mp. [mp=ti, ot, ab, nm, hw] OR health professional.mp. [mp=ti, ot, ab, nm, hw] OR general practitioner.mp. [mp=ti, ot, ab, nm, hw] OR family physician.mp. [mp=ti, ot, ab, nm, hw]) AND influenza.mp. [mp=ti, ot, ab, nm, hw] AND (immunization.mp. [mp=ti, ot, ab, nm, hw] OR

   vaccine.mp. [mp=ti, ot, ab, nm, hw] OR vaccination.mp. [mp=ti, ot, ab, nm, hw] OR

   protection.mp. [mp=ti, ot, ab, nm, hw] OR immunity.mp. [mp=ti, ot, ab, nm, hw] OR

   transmission.mp. [mp=ti, ot, ab, nm, hw] OR immunology.mp. [mp=ti, ot, ab, nm, hw] OR

   (prevention and controle).mp. [mp=ti, ot, ab, nm, hw]) AND (efficacy.mp. [mp=ti, ot, ab, nm, hw] OR effectiveness.mp. [mp=ti, ot, ab, nm, hw]) AND (clinical trial.mp. [mp=ti, ot, ab, nm, hw] OR cohort.mp. [mp=ti, ot, ab, nm, hw]) [↑](#footnote-ref-2)
